# *Trichinella spiralis* antigens prime mixed Th1/Th2 response but do not induce *de novo* generation of Foxp3^+^ T cells *in vitro*

**DOI:** 10.1111/j.1365-3024.2011.01322.x

**Published:** 2011-10

**Authors:** N ILIC, J J WORTHINGTON, A GRUDEN-MOVSESIJAN, M A TRAVIS, L SOFRONIC-MILOSAVLJEVIC, R K GRENCIS

**Affiliations:** 1 Institute for the Application of Nuclear Energy, University of BelgradeBelgrade, Serbia; 2 Immunology Research Group, Faculty of Life Sciences, University of ManchesterManchester, UK

**Keywords:** dendritic cells, Foxp3^+^ Treg cells, Th1/Th2 polarization, *Trichinella spiralis*

## Abstract

Many parasitic helminth infections induce Th2-type immune responses and engage the regulatory network. In this study, we specifically investigated the influence of antigens derived from different life stages of the helminth *Trichinella spiralis* on the polarization of naive CD4^+^ T cells by dendritic cells. Results obtained from C57BL/6 mice showed that *T. spiralis* derived antigens have the capacity to induce bone marrow-derived dendritic cells to acquire an incompletely mature phenotype that promotes a significant proliferation of naive CD4^+^ T cells and a mixed Th1/Th2 cytokine profile with the predominance of Th2 cytokines. Increased production of IL-4, IL-9, IL-10 and IL-13 accompanied increased IFN-γ. Furthermore, dendritic cells pulsed with *T. spiralis* antigens did not induce an increase in the population of Foxp3^+^ T regulatory cells. Although other helminth antigens have demonstrated the capacity to induce *de novo* generation of Foxp3^+^ T regulatory cells, here our *in vitro* studies provide no evidence that *T. spiralis* antigens have this capacity.

## Introduction

Infection with helminth parasites can alter the host’s immune response (1,2). In order to maintain their life cycle, helminths have to modulate the host immune response in a fashion that would enable their long-term survival in the host organism (3). Helminth infections have been shown to potently induce a Th2-type immune response (4–6). The association of helminths and their products with a Th2-type response supports the hypothesis that a Th2 response is protective against helminth parasites and beneficial for the host (7,8). It has also been suggested that helminth parasites have the ability to reduce Th1- as well as Th2-mediated responses by strongly promoting immunoregulatory pathways of the immune response (9,10). A very important role in maintaining immune homeostasis is ascribed to regulatory T cells (Treg), because they have the ability to actively suppress the immune response (11,12). Various populations of regulatory T cells contribute to the maintenance of this equilibrium and establishment of controlled immune responses (13). Treg cells are divided into two main subsets: ‘natural’ CD4^+^ CD25^+^ Foxp3^+^ Treg cells, which develop in the thymus and ‘inducible’ Treg cells, e.g. iTreg that are Foxp3^+^ and Tr1 or Th3 cells that are Foxp3^−^. They develop in the periphery from conventional naive CD4^+^ T cells upon exposure to antigen. Both types of Treg cells are engaged in the response to various infections including a variety of helminths. The nature of Treg-mediated suppression still remains controversial and requires further investigation (14).

Among the proposed mechanisms by which helminths modulate the immune system, one is based upon their ability to alter the maturation of dendritic cells (DCs) (15). Dendritic cells represent a very important link between innate and adaptive immunity, and they are influenced by various pathogens to orchestrate the immune response (16). A number of *in vitro* studies have shown that some helminth products, like the excretory-secretory (ES) antigens derived from *Nippostrongylus brasiliensis* (5), the soluble egg antigen (SEA) of *Schistosoma mansoni* (17) and the ES-62 glycoprotein of *Acanthocheilonema vitae* (18), can induce Th2 immune responses via DCs. On the other hand, helminth antigens like the ES and adult products of *Heligmosomoides polygyrus bakeri* do not promote a Th2 response but rather induce Treg cells under similar conditions (11,15).

*Trichinella spiralis* (*T. spiralis*) is a helminth with three life stages, all of them occurring in a single host. After the ingestion of *T. spiralis* infected muscle, the released infective larvae (L1) undergo the maturation process to the adult reproductive stage within the intestine. Adult parasites produce newborn larvae that migrate to skeletal muscle, where they develop to the L1 stage and trigger differentiation of muscle cells into a so-called nurse cell. Encysted larvae can remain within nurse cells for many years (19). Each *T. spiralis* life stage is characterized by the production of distinctive antigens, and each of these may influence the immune response of the host in its own way. Infection with *T. spiralis* is accompanied by the accumulation of FoxP3^+^ Tregs in the infected muscles during the chronic phase of infection (14). Except for this report, there are no data on the role of Foxp3^+^ Treg cells during the immune response provoked by *T. spiralis* and there is a lack of information concerning the ability of different *T. spiralis* antigens to induce the generation of Foxp3^+^ Treg cells via DCs *in vitro*. The aim of this work was to investigate the influence of antigens from different life stages of *T. spiralis* on DC maturation and T cell polarization *in vitro* as well as their capacity to influence existing and *de novo* Foxp3^+^ T cell populations. In this paper, we demonstrate that different *T. spiralis* antigens induce mixed Th1/Th2 immune responses *in vitro* via DCs but they do not impact on the existing Foxp3+ cell population or induce *de novo* populations.

## Materials and methods

### Parasites, isolation of different life stages and preparation of antigens

Parasite *T. spiralis*, strain ISS161, was maintained by serial passage on Wistar rats at the Institute for the Application of Nuclear Energy (healthy animals were bred at the Institute for medical research, Military Medical Academy, Belgrade, Serbia).

*Trichinella spiralis* infectious muscle larvae (L1) were recovered from infected Wistar rats by a modified method described by Gruden-Movsesijan *et al.* (20). Briefly, digestion of carcasses was performed in prewarmed digestion fluid (1% pepsin in 1% HCl, pH: 1.6–1.8) for 45 min at 45°C with constant stirring. Muscle larvae were then allowed to sediment. The pepsin-HCl solution was removed by aspiration and L1 infective larvae were washed with saline.

Excretory-secretory antigens were collected from L1 muscle larvae cultivated in complete DMEM medium (Sigma Aldrich Gmbh, Steinheim, Germany) supplemented with 10 mm HEPES, 2 mm L-glutamine, 1 mm Na-pyruvate and 50 U/mL pen/strep. Culture fluid was harvested after 18–20 h, filtered through a 0.2 μm filter, concentrated and stored at −20°C.

Muscle larvae crude extract (MLCr) was prepared by sonification of L1 larvae, resuspended in phosphate buffer saline (PBS), on a Potter-Elvehem tissue homogenizer, with constant cooling, until the cuticle was disrupted. The resulting suspension was centrifuged at 20 000 ×*g* for 30 min at 4°C. Supernatant was dialysed in PBS, pH 7, 2. and stored at −20°C.

High mannose component antigen (HMC-Ag) was prepared from MLCr using a concanavalin A-agarose column (ICN Biomedicals, Irvine, CA, USA) equilibrated by 0.1 m acetate buffer, pH 6. MLCr diluted in PBS, with final concentration of 1 mg/mL, was bound to the column for 2 h. Fractions enriched with mannose were evaluated with 0.2 mα-methilmanoside (Sigma Aldrich). Fractions with maximal protein content were joined, dialysed in PBS and stored at −20°C.

Excretory-secretory products of adult *T. spiralis* were obtained according to the procedure described by Gamble (21). Wistar rats, 4–5 months old, were infected with 15 000 L1 larvae, killed 6 days after infection, and adult parasites were isolated from their intestine. Intestine were cut longitudinally and transversely into 2–3 cm pieces, washed in cold PBS and incubated on a mesh at the top of conical dish filled with Hanks balanced salt solution (HBSS) for 3 h at 37°C. Adult parasites were sedimented on the bottom of the dish and afterwards incubated in complete DMEM (Sigma Aldrich) enriched with 10 mm HEPES, 2 mm L-glutamine, 1 mm Na-pyruvate and 50 U/mL pen/strep, for 20 h at 37°C in a humidified incubator. After cultivation, adult parasites were separated from newborn larvae (NBL) by spontaneous sedimentation in conical tubes. NBL were isolated by centrifugation on 400 × g for 10 min and treated with sodium salt of deoxycholic acid in an ultrasonic homogenizer. Soluble fractions of NBL were separated from insoluble ones by centrifugation at 20 000 ×*g*, for 20 min at 4°C. Medium with adult ES products was filtered through a 0.22 μm membrane (Millipore, Billerica, MA, USA) and concentrated on an AMICON Ultrafiltration system (Millipore). All protein concentrations were determined by spectrophotometry and stored at −20° C. All *T. spiralis* antigens preparations were LPS free (demonstrated via Polymyxin B test).

### Generation of bone marrow-derived DCs

Bone marrow-derived DCs (BMDCs) were generated from 8-week-old female wild-type C57BL/6 mice. Mice were maintained at the animal facility of the Faculty of Life Sciences, University of Manchester, UK, under specific pathogen-free conditions.

BMDCs were produced according to the procedure described by Lutz *et al.* (1999) with slight modifications. Briefly, bone marrow was collected from the femurs of C57BL/6 mice and passed through a 100 μm Falcon cell strainer (BD Biosciences, Bedford, MA, USA) to remove debris and clumps. Red blood cells were lysed by Ack buffer (Sigma Aldrich) and remaining cells were washed in culture medium – complete RPMI 1640 (PAA Laboratories Gmbh, Pasching, Austria) supplemented with 2% FCS (PAA Laboratories Gmbh), 10 Mm HEPES (Sigma Aldrich), 2 mm L-glutamine (Gibco, Carlsbad, CA, USA), 100 U/mL penicilin, 100 μg/mL streptomycin and 50 μm 2-mercaptoethanol (Sigma Aldrich). Cells were resuspended at 2 × 10^6^ cells per Petri dish (100 mm) in 20 mL of complete RPMI 1640 medium supplemented with 10% FCS and mouse recombinant GM-CSF (40 ng/mL) (eBioscience, San Diego, CA, USA). Cells were maintained at 37°C in a humidified CO_2_ incubator and were fed on days 3 and 6. On day 8 of cultivation, nonadherent and low-adherent cells were harvested and purified on a LS MACS column using anti-CD11c magnetic beads (Miltenyi Biotec, Auburn, CA, USA). The purity of the obtained cell population was 98% CD11c+, as determined by FACS analyses after staining with FITC labelled anti-mouse CD11c antibodies (eBioscience). These cells were used as immature BMDCs for the experiments.

### *In vitro* BMDCs stimulation assay

Immature BMDCs were cultivated in 6-well plates at 5 × 10^6^ cells/well in 3 mL of complete RPMI 1640 medium enriched with 10% FCS (PAA laboratories Gmbh) and 40 ng/mL mouse recombinant GM-CSF (eBioscience). BMDCs were cultured in medium alone (control group), in the presence of 200 ng/mL *Escherichia coli* LPS (Sigma Aldrich) or they were pulsed with 50 μg/mL of different *T. spiralis* antigens (crude muscle larvae antigen – MLCr; high mannose components antigen (HMC-Ag); excretory-secretory products of L1 muscle larvae – ES L1; excretory-secretory products collected from adult parasites – AdES; or a soluble extract of newborn larvae – NBLAg) for 48 h. At day 10, supernatants were collected for cytokine quantification by Cytometric Bead Array (CBA) and cells were harvested for analyses of co-stimulatory marker expression by FACS and for co-cultivation with T lymphocytes.

### Co-cultivation of CD4^+^ T cells with BMDCs

For evaluation of T cell proliferation by BMDCs, CD4^+^ T cells were isolated from mesenteric lymph nodes of naive C57BL/6 mice and purified by positive selection using anti-CD4 magnetic beads (Miltenyi Biotec) following the manufacturer’s instructions. The purity was 93% CD4^+^ cells, as determined by FACS analysis. Purified CD4^+^ T cells were labelled with 5 μm carboxyfluorescein diacetate succinimidyl ester – CFSE (Invitrogen) and resuspended at 2.5 × 10^6^/mL. Previously harvested BMDCs (nonstimulated and stimulated with LPS or *T*. *spiralis* antigens) were resuspended at 5 × 10^5^/mL. CD4^+^ T cells and BMDCs were then seeded into 48-well plates to obtain a final T cell/BMDCs ratio of 5:1. Co-cultures were incubated for 48 h at 37°C, and after that, 20 U/mL of mouse recombinant IL-2 (Peprotech Inc., Rocky Hill, NJ, USA) was added. Following an additional incubation of 72 h, cells were harvested, washed, resuspended in fresh medium and seeded into 48-well flat-bottom cell culture plate coated with 3 μg/mL anti-mouse CD3 antibody (BD Pharmingen). After 48 h of incubation at 37°C, supernatants were collected for the quantification of cytokines and cells were harvested for the FACS analyses of T cell proliferation and phenotype.

### Cytokine quantification

The cytokine profile of BMDCs was estimated by measuring IL-10, IL-12p70, IL-6 and TNF-α in BMDCs culture supernatants. The capacity of BMDCs to polarize a T cell response was determined according to the cytokine production of CD4^+^ T cells after co-cultivation with stimulated or nonstimulated BMDCs. The cytokine profile of T cells was estimated by measuring IL-4, IL-9, IL-10, IL-13 and IFN-γ in cell culture supernatants. All above-mentioned cytokines were quantified using a CBA test (BD Biosciences) following manufacturers instructions.

### Phenotypic characterization of BMDCs and T cells

For cell surface staining, 1 × 10^6^ cells were washed and treated with Fcγ-blocking reagent (eBioscience) in FACS buffer (PBS with 0.1% BSA and 0.05% NaN_3_) for 30 min on ice. BMDCs were then incubated with the following anti-mouse antibodies: FITC-conjugated anti CD11c, PE-conjugated anti CD80, CD86 and OX40L, APC-conjugated anti MHC II, biotin-conjugated anti ICAM 1 (all from eBiosciences).

To characterize the phenotype of CD4^+^ T cells, cells were surface stained as described above using the following anti-mouse antibodies: FITC-labelled anti CD4 and PE-labelled anti CD25 (both purchased from eBioscience). After washing, intra-cellular staining was performed using Foxp3 Staining Buffer Set and APC labelled anti-mouse Foxp3 antibody (all from eBioscience). Flow cytometry was performed on a FACSCalibur (BD Bioscience, Mississauga, Canada). At least 10 000 gated events were acquired per sample, and data analysis was performed with FlowJo software.

### Treg conversion assay

In order to check the potential of different *T. spiralis* antigens to induce *de novo* generation of Foxp3^+^ T cells mediated by dendritic cells in *in vitro* conditions, a Treg conversion assay was performed. Splenocytes were isolated from spleens of OT-II/Rag1^−/−^ mice, an OVA-specific TCR-transgenic strain. Mice were maintained at the animal facility of the Faculty of Life Sciences, University of Manchester, UK, under specific pathogen-free conditions. Splenocytes were treated with Fcγ-blocking reagent (eBioscience) and afterwards stained with 3 μg/mL of FITC labelled anti-mouse CD4 and PE labelled anti-mouse CD25 antibodies (eBioscience) for 30 min on ice. After washing, the CD4^+^ CD25^−^ cell population was sorted on a FACS Aria (BD Bioscience). The purity was 99% CD4^+^ CD25^−^ cells, as determined by FACS analysis.

Previously harvested BMDCs (nonstimulated and stimulated with LPS or *T*. *spiralis* antigens) were seeded in 96-well round-bottomed cell culture plates at 2.5 × 10^3^ cells/well. CD4^+^ CD25^−^ T cells were added at 5 × 10^4^ cells/well to obtain a final CD4^+^ CD25^−^ T cell/BMDCs ratio of 20:1. OVA (323–339; Sigma-Aldrich) was added in each well to a final concentration of 0.2 μg/mL. Co-cultivated cells were divided into three groups treated with: 40 μg/mL anti-TGF-β (1D11) antibody as negative control (R&D Systems, Minneapolis, MN, USA), 2 ng/mL TGF-β as positive control (R&D Systems) or 40 μg/mL of control mouse IgG (Sigma Aldrich). On days 1 and 3 of cultivation, rhIL-2 (Peprotech) was added in each well to a concentration of 5 ng/mL. On day 4, cells were harvested and surface stained for CD4 and CD25, as well as intra-cellular stained for Foxp3 (eBioscience; according to manufacturer’s protocol), as described above. Flow cytometry was performed on a FACSCalibur (BD Bioscience). At least 10 000 gated events were acquired per sample, and data analysis was performed with FlowJo software.

### Statistical analysis

Data are presented as mean ± SEM. Statistical analyses were performed using Student’s unpaired, two-tailed *t*-test or one-way anova as appropriate.

## Results

### Antigens from different life stages of *T. spiralis* induce incomplete maturation of BMDCs

**Figure 1:**
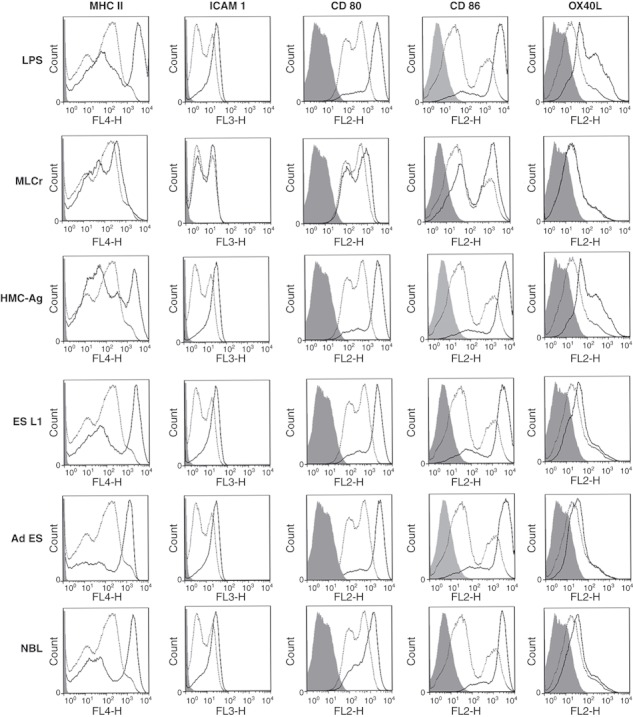
Expression of surface markers on mouse BMDCs pulsed with LPS and *Trichinella spiralis* antigens for 48 hrs (full dark line) as compared with untreated cells (dotted line). Isotype controls are shown with light grey shading. Results are representative of five experiments, each of which gave similar results.

In order to assess whether pulsing BMDCs with different *T. spiralis* antigens alters maturation, nonstimulated BMDCs and BMDCs stimulated with LPS (stimuli that induces complete maturation of DCs and subsequent Th1 polarization in T cells) or *T. spiralis* antigens were analysed for the expression of surface markers and secretion of cytokines. Based on the expression of the surface markers MHC II, ICAM 1, CD80, CD86 and OX40L on CD11c^+^ cells, the data indicated that none of the *T. spiralis* antigen preparations induced the full maturation of BMDCs. While LPS induced full maturation of BMDCs, defined by a significant increase in the expression of MHC II and all co-stimulatory molecules, all the *T. spiralis* antigens induced only a moderate increase in the expression of MHC II, a significant increase in the expression of CD80 and CD86 and a nonsignificant increase in the expression of ICAM 1 and OX40L. In contrast, the mannose enriched derivate of muscle larvae antigen (HMC-Ag) induced the complete maturation of BMDCs, similar to the effect observed with LPS (Figure 1, Table 1).

**Table 1:** Expression of the surface markers on mouse BMDCs: untreated cells, cells pulsed with LPS (positive control) or with different *Trichinella spiralis* antigens for 48 h. Results were obtained by flow cytometry and represent a mean value ± SEM of the results from five independent experiments, performed in triplicates. Mean fluorescent intensity (MFI) and per cent of cells positive for the different surface markers (% pos). ^*^P < 0.05, ^*^^*^P < 0.01, ^*^^*^^*^P < 0.001 represent statistically significant difference to the nonstimulated BMDCs (cultured in medium)

	MHC II		ICAM 1		CD80		CD86		OX40L	
	
	% pos (SEM)	MFI (SEM)	% pos (SEM)	MFI (SEM)	% pos (SEM)	MFI (SEM)	% pos (SEM)	MFI (SEM)	% pos (SEM)	MFI (SEM)
Non-pulsed DCs (Medium)	27 (2)	2387 (108)	245 (2)	14 (1)	34 (2)	801 (38)	31 (1)	667 (36)	22 (2)	136 (7)
DCs pulsed with LPS	60 (3)***	4068 (128)***	35 (7)*	19 (2)*	61 (6)***	1621 (42)***	60 (4)***	1294 (75)***	35 (7)*	176 (23)*
DCs pulsed with MLCr	34 (4)*	2885 (280)*	27 (3)	16 (2)	40 (4)*	969 (104)*	37 (3)*	779 (52)*	26 (2)*	164 (17)*
DCs pulsed with HMC-Ag	57 (7)***	4237 (275)***	33 (4)*	18 (3)*	57 (6)***	2667 (265)***	60 (4)***	1932 (165)***	30 (4)*	180 (23)*
DCs pulsed with ES LI	44 (4)**	3221 (211)**	25 (4)	17 (2)	55 (3)***	1592 (84)***	54 (3)***	1095 (109)***	24 (2)	157 (27)
DCs pulsed with Ad ES	33 (4)*	3027 (332)*	28 (2)	15 (2)	48 (3)***	1710 (101)***	60 (5)***	1146 (122)***	25 (2)	143 (18)
DCs pulsed with NBL	32 (3)*	2939 (254)*	28 (3)	16 (2)	58 (4)***	1608 (70)***	49 (5)**	1078 (128)**	25 (2)	141 (27)

### BMDCs pulsed with *T. spiralis* antigens produce both pro- and anti-inflammatory cytokines

**Figure 2:**
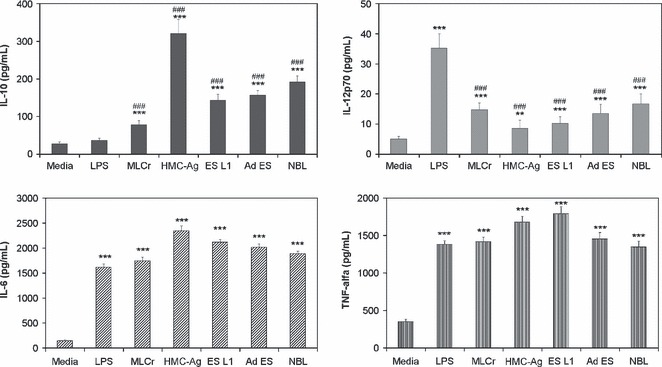
Production of IL-10, IL-12p70, IL-6 and TNF-α by BMDCs cultivated in medium alone (control), LPS or *Trichinella spiralis* antigens. Cytokine levels were measured in cell culture supernatants using a Cytometric Bead Array. Data are means ± SEM of one experiment carried out in triplicate, representative of four independent experiments. **P < 0.01 ***P < 0.001 represents statistically significant difference to the BMDCs cultured in medium. ###*P* < 0.001 represents statistically significant difference to the LPS-stimulated BMDCs.

In addition to determining the expression of surface markers on BMDCs, we measured the cytokines produced by these cells in response to different *T. spiralis* antigens, in comparison with medium or LPS-treated BMDCs. Cytokine profiles of the *T. spiralis* stimulated BMDCs (Figure 2) showed that all applied *T. spiralis* antigens induced significantly elevated production of the pro-inflammatory cytokines IL-12p70, IL-6 and TNF-α, compared to DCs cultivated in medium alone. However, the production of IL-12p70 in response to *T*. *spiralis* antigens is significantly lower compared to LPS-stimulated production. Parasite antigens also induced a high increase in the anti-inflammatory cytokine IL-10 compared to controls (nonstimulated BMDCs and LPS-stimulated DCs).

### *Trichinella spiralis* antigens induce the proliferation of naive CD4^+^ T cells mediated by BMDCs

**Figure 3:**
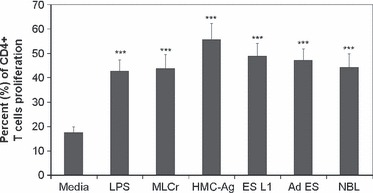
Proliferation of CD4^+^ T cells cultured with stimulated or non-stimulated BMDCs. CD4^+^ T cells were isolated from mesenteric lymph nodes of C57BL/6 mice, labeled with CFSE and co-cultured for 7 days with BMDCs (non-stimulated, stimulated with LPS or *Trichinella spiralis* antigens) in the presence of recombinant mouse IL-2 and anti-CD3 antibody. Data are means ± SEM of one experiment carried out in triplicate representative of four independent experiments and they are expressed as the percentage of proliferating cells. ***P < 0.001 represents statistically significant difference to the non-stimulated BMDCs.

The capacity of *T. spiralis* stimulated BMDCs to induce the proliferation of T cells was investigated by co-cultivation of BMDCs pulsed with different *T. spiralis* antigens and naïve CD4^+^ T cells labelled with CFSE (Figure 3). BMDCs stimulated with *T. spiralis* antigens induced a 2–3-fold increase in the percentage of proliferating cells compared to controls (nonstimulated BMDCs).

### Mixed Th1/Th2 polarization of CD4^+^ T cells activated by BMDCs pulsed with *T. spiralis* antigens

**Figure 4:**
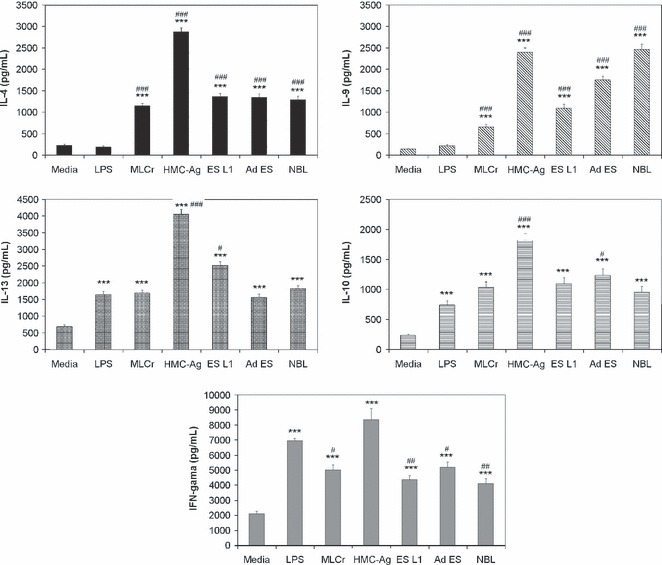
Production of IL-4, IL-9, IL-13, IL-10 and IFN-γ by purified CD4^+^ T cells, stimulated with BMDCs pulsed with different *Trichinella spiralis* antigens. CD4^+^ T cells were isolated from mesenteric lymph nodes of naive C57BL/6 mice and co-cultured for 7 days with BMDCs (non-stimulated and stimulated with LPS or *T*. *spiralis* antigens) in the presence of recombinant mouse IL-2 and anti-CD3 antibody. Data are mean value ±SEM of triplicates in Cytometric Bead Array. Results represent one out of four independent experiments. ***P < 0.001 represents statistically significant difference to the non-stimulated BMDCs. #*P* < 0.05, ##*P* <0.01, ###*P* < 0.001 represents statistically significant difference to the LPS-stimulated BMDCs.

The potential of different *T. spiralis* antigens to induce BMDCs to polarize the immune response of co-cultured CD4^+^ T cells was examined by the cytokine profile of co-cultured cells (Figure 4). Results showed that all applied *T. spiralis* antigens induced a mixed Th1/Th2 profile. The production of the Th2 cytokines IL-4, IL-9, IL-13 and IL-10 was highly increased compared to nonstimulated BMDCs. As for the Th1 cytokine, IFN-γ, all applied *T. spiralis* antigens provoked significant production of this cytokine compared to nonstimulated BMDCs. Nevertheless, this production was much lower than the production of IFN-γ provoked by LPS-stimulated BMDCs. LPS-stimulated BMDCs did affect the production of IL-10 and IL-13, but had no effect on IL-4 and IL-9. The results clearly show that *T. spiralis* antigens induce highly elevated production of the typical Th2 cytokines IL-4 and IL-9 compared to the production induced by LPS.

### Influence of BMDCs stimulated with *T. spiralis* antigens on CD4^+^ CD25^+^ T regulatory cell population

**Figure 5:**
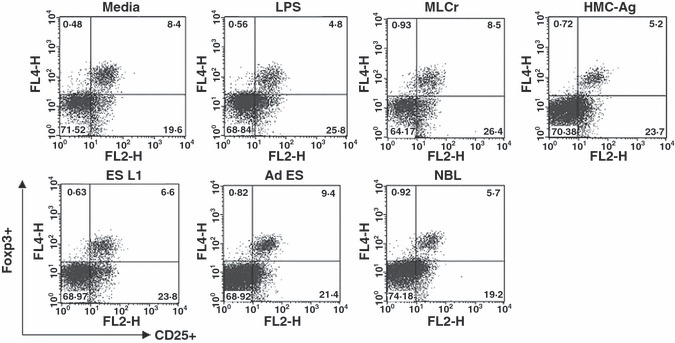
Foxp3^+^ cells within the CD4^+^ population of T cells isolated from mesenteric lymph nodes of C57BL/6 mice cultured with BMDCs (non-stimulated and stimulated with LPS or *Trichinella spiralis* antigens) for 7 days in presence of recombinant mouse IL-2 and anti-CD3 antibody. Results are representative of four experiments, each of which gave similar results.

In order to evaluate the potential of *T. spiralis* antigens to expand/induce Foxp3^+^ T cells *in vitro,* we measured the percentage of CD4^+^ CD25^+^ Foxp3^+^ T regulatory cells in the resulting population of CD4^+^ cells from C57BL6 mice after cultivation with BMDCs stimulated with *T. spiralis* antigens compared to controls (CD4^+^ cells cultivated with nonstimulated BMDCs or stimulated with LPS). The obtained results showed that LPS does not increase the percentage of Foxp3^+^ cells. At the same time, none of the applied *T. spiralis* antigens induced the elevation in the percentage of Foxp3^+^ T cells (Figure 5).

**Figure 6:**
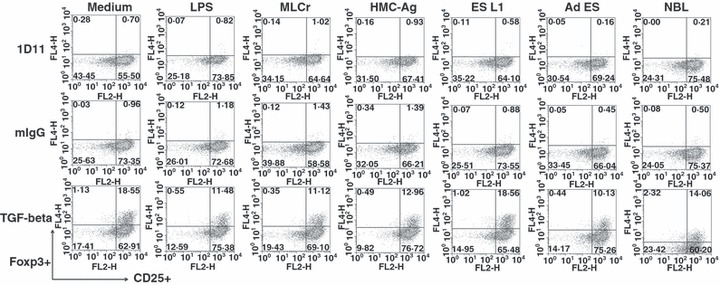
Percentage of CD4^+^ CD25^+^ Foxp3^+^ cells in the resulting cell population after co-cultivation of CD4^+^ CD25^−^ cells from OTII/Rag1^−/−^ mice with BMDCs pulsed with different *Trichinella spiralis* antigens or LPS in the presence of 1D11 antibody (negative control), TGF-β (positive control) or control mouse IgG. Results are representative of four experiments, each of which gave similar results.

Because Foxp3^+^ T cells represent only a small percentage of the total T cell population, it is hard to distinguish whether *T. spiralis* antigens affect the Foxp3^+^ population of cells in *in vitro* conditions when stimulated BMDCs were co-cultured with the complete CD4^+^ T cell population. In order to estimate their impact on *de novo* induction of Foxp3^+^ expression on T cells, BMDCs stimulated with *T. spiralis* antigens were cultured with CD4^+^ CD25^−^ T cells isolated from OTII mice on a Rag1^−/−^ background (which contain no Tregs). Cells were cultured in the presence of anti-TGF-β antibody (1D11) as a negative control, TGF-β as a positive control or control mouse IgG. The results showed that none of the applied *T. spiralis* antigens induced *de novo* expression of Foxp3 on T cells (Figure 6). In the group of cells cultivated in the presence of TGF-β, which potently induces Foxp3^+^ cells, all *T. spiralis* antigens except ES L1, decreased the percentage of Foxp3^+^ T cells compared to the control (medium) in a similar manner to LPS. The results clearly show that antigens from all *T. spiralis* life stages do not induce *de novo* Foxp3 expression in T cells under *in vitro* conditions and the majority actually reduce Treg induction.

## Discussion

The maturation status of DCs, characterized by the expression of surface markers and cytokine production, has a major impact on the polarization of the immune response triggered by pathogens. There is evidence that the activation of DCs in response to parasitic helminth antigens leads to incomplete maturation (22,23). Results obtained in this study examining the effect of *T. spiralis* antigens derived from the different life stages of the parasite, showed that none of these *T. spiralis* antigens tested (MLCr, ES L1, Ad ES, NBL) induced the complete maturation of mouse BMDCs. These results correlate with our previous results obtained in a rat model system (24) and those obtained by authors who investigated the impact of antigens derived from *S. mansoni* (17,25–27), *N. brasiliensis* (5) or *A. suum* (22) on DC maturation. Langelaar *et al.* (28) showed that *T. spiralis* excretory-secretory antigens completely abolished the surface marker expression of *E. coli* LPS stimulated DCs, but did not affect DC maturation induced by LPS from *Neisseria meningitides*.

As it was described that changes in carbohydrate structure can influence the capacity of antigens to induce the maturation of DCs and subsequently to polarize the immune response (29), we performed the partial purification of MLCr and created a concentrated antigen with high mannose content (HMC-Ag). Here, we show that this *T. spiralis* antigen was able to induce the complete maturation of mouse DCs, similar to the effect observed with LPS. The ability of purified and concentrated components with high mannose content to induce a different maturation profile compared to native *T. spiralis* antigens, could be explained by the fact that multimerization of ligands, as well as their concentration, influence the number of engaged receptors and cross-linking, and this has a great impact on the intensity and duration of signalling cascades in activated DCs (30).

Besides the expression of DC surface markers, cytokine production by *T. spiralis* stimulated DCs presents an important signal for the initiation and regulation of immune responses. All applied *T. spiralis* antigens induced an increased production of pro-inflammatory (IL-6, IL-12p70 and TNF-α) as well as anti-inflammatory (IL-10) cytokines by BMDCs. This result does not correlate with previous findings that claimed that the elevated levels of IL-10 suppress the production of IL-12p70 (31,32). However, there are many pathogens that induce both pro- and anti-inflammatory cytokines to different extents but they all result in a Th2 or mixed Th1/Th2 responses (33–35). The proliferation assay showed that DCs stimulated with different *T. spiralis* antigens have the capacity to present those antigens to naive T cells, e.g. DCs whether matured (pulsed with HMC-Ag) or partially matured (pulsed with MLCr, ES L1, Ad ES, NBL) induce significantly elevated proliferation of CD4^+^ T cells compared to controls. This correlates with the results of other authors who showed that parasite antigens, derived from *L. brasiliensis* (33) and *L. major* (36), induce incomplete DC maturation, but that these DCs are capable of presenting parasitic antigens to naive T cells and induce significant proliferation.

The impact of *T. spiralis* antigens on the polarization of immune responses, mediated by DCs, was determined according to the cytokine profile created by CD4^+^ T cells co-cultivated with *T. spiralis* stimulated BMDCs. All applied *T. spiralis* antigens induced an increase in Th2 cytokines IL-4, IL-9 and IL-13, the Th1 cytokine IFN-γ and also IL-10. The antigen with enriched mannose content, HMC-Ag, induced the most prominent increase in the production of all investigated cytokines compared to the control. HMC-Ag had a greater impact on the production of IFN-γ even compared to the effect of LPS that is a well known Th1 stimulus. This result implies that this mixture of antigens, purified according to carbohydrate composition, contains antigens rich in sugars and able to trigger different arms of the immune response as a reaction to *T. spiralis*. The obtained results indicate that all investigated *T. spiralis* antigens induce a mixed Th1/Th2 cytokine profile with the predominance of Th2 cytokines. *T. spiralis* antigens induced a highly significant elevation of Th2-specific cytokines, e.g. IL-4 and IL-9 compared to the effect of LPS (Th1 stimulus). As it is known that IL-9 is a potent Th2 adjuvant and has an important role in the induction of a Th2-type response (37) and that IL-4 is important for the establishment and maintenance of a Th2 response, results presented in this study indicate that *T. spiralis* antigens from all three life stages of the parasite contribute to the development and maintenance of a Th2 response.

According to the high amounts of IL-10 produced by CD4^+^ T cells after the co-cultivation with *T. spiralis* stimulated BMDCs, it could be presumed that Treg cells are present in the resulting population of T cells. There are a number of results showing the importance of Treg cells in modelling the immune response provoked by parasites (9,38). Some chronic parasite infections (like those with *Heligmosomoides polygyrus bakeri*, *Schistosomamansoni* and *Acanthocheilonema. vitae*) induce a regulatory response via an increase in the proportion of CD4^+^ CD25^+^ Foxp3^+^ T cells besides Th2 responses (38,39).

Our results showed that *T*. *spiralis* antigens do not induce an increase in the number of CD4^+^ CD25^+^ Foxp3^+^ T cells *in vitro*. The disproportion between the result obtained for the percentage of Foxp3^+^ cells and one considering the level of IL-10 produced by stimulated CD4^+^ T cells could be explained by the fact that IL-10 can be the product of Foxp3 T regulatory cell populations (13,40) or by Th2 cells (41).

In order to check whether antigens derived from different life stages of *T. spiralis* have the ability to induce *de novo* generation of Foxp3^+^ T cells *in vitro*, we performed the co-cultivation of CD4^+^ CD25^−^ T cells isolated from OT II mice, on a Rag1^−/−^ background, with *T. spiralis* stimulated BMDCs. The results clearly show that antigens from all *T. spiralis* life stages do not readily induce Foxp3 expression in T cells *in vitro*. In fact, *T. spiralis* antigen preparations appear to reduce Foxp3 conversion in response to TGF-β. It is known that the Th1 and Th2 cytokines IFN-γ and IL-4 are sufficient to block Foxp3^+^ induction (42,43) and so, because the applied antigens provoked an increased production of IL-4 and IFN-γ, the presence of these cytokines could have prevented the induction of Foxp3^+^ Treg cells. Unlike our results, it has been recently shown that ES antigens derived from *Heligmosomoides polygyrus bakeri* and *Teladorsagia circumcincta* have a TGF-β mimicking effect and are capable of inducing *de novo* generation of Foxp3^+^ Treg cells (11). The authors did not observe this phenomenon with ES antigens derived from *Haemonchus contortus* and *Nippostrongylus brasiliensis,* which suggests that release of TGF-β ligands is not a general feature of all helminth parasites. One possible explanation of differences in Treg induction between parasites is the difference in life cycles of the nematodes. *Heligmosomoides polygyrus bakeri* adult worms benefit from Treg induction and a suppressed immune response as a chronic infection allows an increase in intestinal egg production while minimizing host pathology. Alternatively, adult *T. spiralis* worms require only a short period of time in the intestine to produce newborn larvae that require a period of around 15 days to develop into nurse cells allowing the life cycle of the parasite to continue upon death of the host. Too many newborn larvae can result in extensive pathology and death, before nurse cell formation is complete, thus preventing the completion of the life cycle. Therefore, Treg induction is not beneficial to *T*. *spiralis* during the intestinal phase as it prefers a strong immune response to indirectly control the magnitude of the newborn larvae released systemically to prevent potential host death. Treg cells were observed by other authors (14) during the muscle phase of the infection, after newborn larvae have established, which suggests that it is beneficial for the survival of the parasite during the nurse cell phase of infection. Our results *in vitro* with *T. spiralis* antigens from all life stages including muscle larvae antigens did not correlate with the above-mentioned results obtained *in vivo*. The reason for that could be that the whole network of different mediators present *in vivo* may contribute to the increase in the Foxp3^+^ cell populations in the infected host, but these conditions could not be reproduced *in vitro*. DCs may respond to helminth infection by sensing stress signals or tissue damage inflicted by helminths or their products. The presence of these signals during infection can induce such modulation of DCs that would consequently lead to a fine-tuned immune response consisting of Th2 and Treg responses *in vivo*. In conclusion, we demonstrated that BMDCs exposed to *T. spiralis* antigens from different life stages of the parasite lack the conventional characteristics required for complete DC maturation. Nevertheless, as expected, they clearly possess the capacity to present parasite antigens to naïve T cells. Immune responses provoked by *T. spiralis* stimulated BMDCs *in vitro* could not be described as ‘classical’ Th2 and Treg responses, and it rather represents a mixed Th1 and Th2 response. In the described conditions, BMDCs cultivated with *T. spiralis* antigens can induce neither the expansion of natural Treg cells present in the population of naïve T cells nor *de novo* expression of Foxp3 in T cells.
